# Bringing the patient voice into the operating room: engaging patients in surgical safety research with the Operating Room Black Box^®^

**DOI:** 10.1186/s40900-022-00367-5

**Published:** 2022-07-23

**Authors:** Cole Etherington, Maxime Lê, Laurie Proulx, Sylvain Boet

**Affiliations:** 1 Clinical Epidemiology Program, Ottawa Hospital Research Institute, Ottawa, Canada; 2 Department of Anesthesiology and Pain Medicine, University of Ottawa, Ottawa, Canada; 3 The Ottawa Hospital, Ottawa, Canada; 4 Canadian Arthritis Patient Alliance, Ottawa, Canada; 5 Department of Innovation in Medical Education, University of Ottawa, Ottawa, Canada; 6 Francophone Affairs, Faculty of Medicine, University of Ottawa, Ottawa, ON Canada; 7 Institute du Savoir Monfort, Ottawa, ON Canada; 8 Faculty of Education, University of Ottawa, Ottawa, ON Canada; 9 Department of Anesthesiology and Pain Medicine, The Ottawa Hospital, University of Ottawa, 501 Smyth Road, Ottawa, ON K1H 8L6 Canada

**Keywords:** Patient participation, Patient safety, Operating rooms, Quality improvement, Healthcare delivery, Patient engagement, Surgical safety

## Abstract

**Background:**

Surgery is one of the most common patient experiences in the health care system. Yet, efforts to engage patients in surgical safety research have not matched those of other health care fields. This is a critical issue given the nature of surgery inhibits patients’ abilities to advocate for themselves as they are typically under anesthetic when the procedure is performed. We partnered with patients throughout our research program, which uses the Operating Room Black Box^®^ to enhance surgical patient safety through transparent and proactive analysis of human factors to detect and prevent avoidable errors.

**Main body:**

In this article, we outline the need for, and our approach to, patient engagement in surgical safety research. Our approach included a series of planned activities and skill development opportunities designed to build capacity and bring together patients, clinicians, and researchers to inform research and practice. We also conducted evaluation surveys during the first year of our program, which have indicated a positive experience by both patient partners and the research team.

**Conclusion:**

We believe our approach can serve as an important first step toward building a model for patient engagement in the surgical safety field and could significantly contribute to improved quality of care and outcomes for surgical patients.

**Supplementary Information:**

The online version contains supplementary material available at 10.1186/s40900-022-00367-5.

## Plain English summary

Although surgery is one of the most common patient experiences, patients have not been engaged in surgical safety research. Patients were engaged as patient advisors in the use of the Operating Room Black Box^®^ at The Ottawa Hospital. The Operating Room Black Box^®^ is a tool that is used in the operating room (OR) which captures audio, video, patient vital signs and other information that are analyzed for research purposes. The aim of the OR Black Box^®^ is to learn from experience and improve practice and care. Patient advisors influenced the nature of the activities undertaken, co-developed the communication materials for patients, evaluated patient engagement practices, and disseminated the research results. These patient engagement activities are an important first step in engaging patients in surgical safety research.

**Supplementary Information:**

The online version contains supplementary material available at 10.1186/s40900-022-00367-5.

## Introduction

Surgery is one of the most common patient experiences in acute care [1]. Yet, efforts to engage patients in operative safety research have not matched those of other health care fields [2]. This is a critical issue for several reasons. First, anesthesia inhibits patients’ abilities to advocate for themselves as they are sedated during surgery. Second, nearly one in ten surgical patients will experience a complication (e.g. infection, injury, bowel obstruction, pulmonary embolism) [1], which can reduce patients’ quality of life, delay recovery, and sometimes result in mortality [3]. Third, the rate of preventable errors in surgery remains relatively high (> 50%) [3–6], and there has been minimal improvement in recent years despite ongoing proliferation of practice interventions [1, 7]. While interventions for the operating (OR) room have typically not been developed in partnership with patients [8, 9], both evidence and practice demonstrate this is essential for preventing, responding to and learning from patient safety incidents [10].

### ***The OR Black Box***^***®***^*** as a unique opportunity for patient engagement***

Our research team uses OR Black Box^®^ technology [11, 12] to study and improve OR practice and obtain the best possible patient outcomes. Like black boxes used in aviation, the OR Black Box^®^ is an innovative tool that records detailed information from the surgical environment (e.g. audio, video, physiological and environmental data). Data can be used for research purposes and to improve practice. Further information about the OR Black Box^®^ is described elsewhere [13–15]. The Ottawa Hospital was among the first institutions in the world to adopt the OR Black Box^®^, which we successfully implemented in June 2018 [14]. We are currently using this technology to study teamwork in everyday clinical practice, to identify areas for improvement, and to assess the effectiveness of interventions (manuscripts in preparation and under review). Our initial exploratory studies aim to better understand current practices in the OR, which will inform decision-making regarding areas for improvement, future research, and intervention development. Once identified, we will work with our patient partners to prioritize key areas.

Unlike other OR technologies that may be used to collect data, the OR Black Box^®^ provides systematic, real-time capture of a wider range of intraoperative data than has previously been possible [14]. Room cameras, microphones, laparoscopic cameras, along with various devices and sensors capture patient physiology and environmental factors (e.g. decibel level, room temperature) [14]. High definition (HD) cameras record a 180-degree view of the surgical field and HD unidirectional microphones are strategically placed around OR team members (e.g. surgeon, anesthesiologist, scrub nurse) [14].

To ensure this innovation could be optimally beneficial to patients, we determined it would be important to engage them at each stage of OR Black Box^®^ implementation and subsequent research studies. However, there was a lack of examples within the surgical safety literature on how to best engage patient partners. In this commentary, we therefore aim to outline our approach to patient engagement in order to provide an example that may be of use to other research teams in surgery.

## Background

### Why does patient engagement in surgical safety research matter?

As experienced health system users, patients can bring a unique perspective to research that can increase its accountability, relevance, and transparency [16–18]. While patient-centered care is a key underlying principle of modern perioperative practice [19], surgical safety research has often overlooked the role of patients in being active partners and co-designers in the research process rather than solely participants [18]. This is a missed opportunity to increase the value and impact of surgical safety research, particularly as patients are the ultimate recipients of research findings [20]. Given there has not been any substantial annual reduction in patient safety events in recent years despite a proliferation of practice interventions and medical advances [1, 7], it is time to approach research differently.

Surgical patients have first-hand experience of a variety of services throughout the perioperative pathway, including gaps in the current healthcare system. This type of lived experience can be invaluable for informing surgical safety research priorities, analysis of results, and translation of findings into clinical practice [21]. For example, including the patient in OR team briefings (e.g. the patient confirms their name and allergies) when possible has been suggested as a patient-centered practice [19], but its implementation and effects have never been investigated. Involving patient partners could provide key insights as to how patients might prefer to be included in the activities of the OR team while awake (e.g., under regional anesthesia) and reveal specific considerations for when fully anesthetized. By including patient partners, surgical safety research agendas can become more informed and more accountable [20], ultimately enhancing patient-centered surgical care [21, 22].

## Main text

### Our approach to patient engagement

To recruit patient partners, we leveraged the Patient and Family Advisor Council (PFAC) at The Ottawa Hospital (TOH). PFAC maintains an active database of patients and family members who are interested in partnering with hospital researchers, clinicians, or administrators to improve safety and quality of care at TOH. PFAC connected us to potential patient partners. We aimed to recruit two patients rather than one in order to avoid tokenism, help patients to feel more comfortable to participate in team meetings, and ensure a balanced workload. Recruiting two patient partners was also suggested by LP during her interview, based on her vast experience in working with research teams. We also aimed to recruit patients representing different genders and age groups, as experiences of surgery can vary based on these characteristics. We conducted interviews with several candidates. Interviews were approximately 45 to 60 min, and inquired about patients’ previous experiences with both surgery and research, as well as their motivations for and interest in combining the two. We ultimately selected two patient advisors whom we felt had the best fit with our team and who had lived experience of multiple surgeries (ML, LP) to join our team. It was important to us that patients had experience of different types of surgeries given the variation in experiences that may occur across procedures. This would help to inform our implementation of the OR Black Box^®^ in a more generalizable manner which could be relevant to patients regardless of their procedure type.

We consulted with the Strategy for Patient-Oriented Research (SPOR) Program Facilitator of the Ottawa Methods Centre (OMC) to obtain guidance for working with patient advisors. The OMC is a member of the Ontario SPOR Support Unit housed at the Ottawa Hospital Research Institute. The Unit is funded by the Canadian Institutes of Health Research to provide investigators with methodological guidance on patient-oriented research. The OMC SPOR Program Facilitator provided our team with resources on engaging patients in research, which covered a range of topics such as conducting team meetings and activity planning. Based on this guidance, we planned an initial set of patient engagement activities, which were subsequently refined after the patient advisors joined our team (Table 1).

**Table 1:** Patient engagement activities in the OR Black Box^®^ research program

Co-design of terms of reference and organizational onboarding
Grant review
Co-design of communication campaign messages and materials based on results qualitative interview study of patient perceptions
Conference attendance and presentations
Media interviews
Regular evaluation of patient engagement approach
Planning future research

After both patient advisors were recruited, terms of reference were co-designed based on an example template provided by the OMC SPOR Program Facilitator (Additional file 1). We elected to collaboratively develop the terms of reference to establish a clear understanding of roles and responsibilities by all team members. The terms of reference were also designed to ensure that the research partnership would be respectful of patient partners’ other commitments, responsibilities, and needs. This included, for example, setting meetings on particular dates based on patient advisors’ availability and providing travel vouchers as needed. Patient advisors were then formally onboarded at our centre through training and orientation provided by TOH to orient all new volunteers to hospital policies and procedures. We also held an initial team meeting to orient the patient advisors to our research program and invited them to various events at our research institution to learn more about the organization and work being done.

Planned patient engagement activities commenced following patient advisor onboarding and evolved as needed based on advisors’ feedback (Table 1). Below we provide a chronological account and brief description of each activity.

The first planned activity involved grant review to establish our research program and implement the OR Black Box^®^. Feedback obtained at this stage was incorporated into the grant applications and also helped to develop new activities, such as regular evaluation of our patient engagement approach. The second planned activity was co-design of the patient communication campaign as part of the OR Black Box^®^ implementation at our centre. Based on data from our previously conducted study of patient perceptions regarding this technology [23], patient advisors created messages and materials to be distributed to patients prior to surgery. The details of the communication campaign are described elsewhere [14].

In brief, our research question focused on determining what might prevent patients from agreeing to have their surgery captured by the OR Black Box^®^ and what could make them feel more comfortable with it [23]. Results indicated that patients unanimously valued the much-needed transparency offered by this tool and were supportive of its use for patient safety research [23]. From the interviews, we identified key themes regarding important information patients would want to have communicated to them prior to having surgery in the OR Black Box^®^ room [23]. For example, patients expressed that they wanted to be informed about the research goals (i.e., what we were using the technology to study) and also wanted to be told about the privacy measures in place [23]. We incorporated these themes into an evidence-based, hospital-wide communications campaign to inform patients and their families about the OR Black Box^®^.

The communications campaign was developed in collaboration with our patient advisors (ML, LP). The advisors helped to identify the most important themes to share with the larger patient population based on their own experiences of surgery. They then took part in a design session alongside members of the research team to pair messages with relatable images and to develop appropriate and accessible wording. From there, we engaged in an iterative review process of informational poster and pamphlet prototypes. We also worked closely with the communications team at our centre. The final poster and pamphlet were agreed upon by everyone involved and are currently available to patients at our centre. Patient partners also suggested the appropriate timing in the care pathway to receive this information; namely, during the preoperative assessment which takes places several weeks before the procedure. This was suggested because it would give patients enough time to learn and ask questions about the OR Black Box^®^, without adding to any anxiety experienced on the day of surgery. This important consideration may have been overlooked without the involvement of our patient advisors and has been key to the high number of patients agreeing to be recorded during their surgery at our centre.

As the campaign was launched, the patient advisors participated in numerous dissemination activities. Our initial engagement plan was for the patient partners to attend and present their experience with implementing the OR Black Box^®^ for surgical safety research at the annual Surgical Safety Network (SSN) meeting. The SSN meeting is an international meeting of surgical safety experts, including clinician-researchers and organizational leaders. Following this event, however, many additional opportunities arose for patient partners to share their experience. This was not anticipated or planned for ahead of time but was fully embraced and supported by our team. In addition, we did not initially plan for patient partners to be interviewed by the media, but this occurred several times over the course of our project as patient partners expressed interest in these opportunities. The patient advisors shared their experience and insights on patient engagement with diverse audiences, including the public, hospital stakeholders, the broader health research community, and other patient-related organizations. This ultimately facilitated positive momentum around the OR Black Box^®^ initiative, particularly for centres considering implementing the OR Black Box^®^ in the future. Sharing their lived experiences to these audiences thus laid the groundwork for future international collaborations to advance patient engagement in surgical safety research.

Following successful implementation of the OR Black Box^®^ at our centre, we began to plan our first series of research studies. Patient advisors assisted with developing additional grant applications and study protocols, and were listed as co-authors/co-applicants as appropriate. Their feedback was consistently incorporated, often helping to identify key research gaps. For example, our patient partners have shared their experiences of considerable variation in how healthcare professionals interact with patients in the pathway leading up to general anesthesia or during the surgery when performed under sedation or regional anesthesia. This has expanded our research program to include a series of studies that focus on how to develop and implement patient-centered teamwork in every OR. This could involve ensuring that team members explain what is going on, what will happen next, and what choices are available at each point in the process where possible. A patient-centered conception of OR teamwork may also shift patient safety culture and further empower patients to play an active role in their surgical care, even in the OR. With the OR Black Box^®^, we will be able to study the extent to which these things happen, identify key moments for including patients, and determine intervention effectiveness. As we move forward with this work, we will work with our patient partners to evaluate our data and translate our findings into practice.

### Learning from our approach

Throughout the first year of our program development, we conducted three informal evaluation surveys in order to reflect on and improve our approach to patient engagement. Surveys were conducted at months 5, 10 and 15. Patient partners (n = 2) and research team members (n = 4) were surveyed, and results were collated and shared with the full team after each survey. Research Ethics Board approval was not required as the surveys were viewed as internal quality improvement activities. We specifically explored satisfaction with the experience, attitudes toward engagement, feelings of being heard and understood, perceived team effectiveness and trust, resources invested (e.g. time, money), products emerging from our activities, and practice/clinical improvements resulting from our activities. The survey used was recommended by the OMC SPOR Program Facilitator and developed by Patients Canada, a national organization that works to bring the patient voice to healthcare decision-making [24]. Survey questions capture the research team’s and patient partners’ experiences with the initial engagement and subsequent research processes. This was deemed suitable for our purposes of documenting and learning from our experiences to constructively move our research program forward. Of note, this was not a formal program evaluation but rather an internal assessment for the purposes of our team. As our program expands, there may be opportunities to conduct a formal evaluation.

Overall, the surveys indicated a positive experience by both patient partners and the research team. Both patient partners reported that, in their opinion, their insights and comments impacted the decisions of the team regarding the research project. Research team members rated the feedback given by patient partners as very valuable and indicated that patients should definitely be involved in this type of research. Patient partners indicated that our group’s collaboration helped them to learn more about the research process, the challenges involved, and important information to relay to the patient community. Research team members expressed that patients’ voices were assets to the project and that researchers should “never underestimate how much patients can offer”. Patients also reported that the team accommodated their needs appropriately (e.g. flexibility in meeting times and locations), that they felt comfortable speaking up in meetings, and that they felt equipped to contribute to the project.

From the surveys, we identified resource-related issues as a key area for improvement. Research projects are primarily grant-funded, which can be unpredictable and make long-term planning difficult. Our initial year of patient engagement was funded, meaning we were able to provide our patient partners with compensation (payment) and cover travel costs and other related expenses. However, we recommend that funding agencies and healthcare organizations consider strategies to secure funding for sustainable and long-term patient engagement activities. Alternatively, research teams will need to secure additional funds to account for the extended contributions of patient partners. We also suggest budgeting for team-building activities, networking and training opportunities, and unanticipated expenses that may be incurred by patient partners as a result of engaging with the project (e.g., having to take a day off of work to attend conferences).

### Lessons learned

From our experience so far, we have obtained several insights that may be useful to other research teams seeking to engage patient partners in their surgical safety projects. In addition to establishing open communication, respect, and active involvement, which have been emphasized by other authors [20, 25, 26] we identified several key logistical points based on the evaluation survey responses. These lessons are summarized in Table 2. With regard to engagement in surgical safety research in particular, it may be beneficial to seek patient partners with experience of certain procedures depending on the research area. In our case, a wide range of experiences was suitable to inform OR Black Box^®^ implementation; however, researchers focusing on a narrower surgical field (e.g., orthopedics, neurology, cardiovascular) may wish to engage patients who have experienced those procedures specifically.

**Table 2:** Engaging patients in surgical safety research: lessons learned from experience

Engage patient partners early (ideally, in the project planning/grant preparation phase)
Co-design terms of reference, engagement activities, and study protocols
Budget for patient engagement activities and financial support
Recognize patients’ contributions to the research (e.g., authorship, compensation)
Conduct regular evaluations to identify successes and areas for improvement
Plan for the future

### Future directions

Data collection with the OR Black Box^®^ is currently underway. This data will be used to identify areas for improvement in OR teamwork and interventions will be co-designed with patient partners. We plan to assess the impact of OR teamwork and teamwork interventions on patient-centered outcomes, determined in partnership with patient advisors. It is our hope that patient engagement will ensure a direct link between research objectives and outputs and the lived experiences of surgical patients and real-world healthcare services.

We will continue to explore avenues for building capacity in surgical patient engagement across centres in order to maximize research impact and dissemination. We also plan to work with our centre and others to support patient engagement in surgical safety research long-term. For example, we recognize that patient needs, and experiences may vary by type of surgery. Therefore, it may be valuable to form sub-committees within our network where patient partners can be involved in research across different surgical contexts (e.g., emergency vs. elective procedures, oncological, cardiovascular, procedures for chronic disease management, etc.). Innovation and collaboration are needed in this area to ensure enduring and meaningful patient partnerships in research that will ultimately make surgery safer.

## Conclusions

We believe that engaging patient partners in implementation of the OR Black Box^®^ was a key aspect of successful implementation and provided a strong foundation for patient-centered surgical safety research at our centre. Our approach represents an important first step toward advancing patient engagement in the surgical safety field at a larger scale. Funding agencies and healthcare organizations should consider strategies to support meaningful and long-term collaborations between researchers and patient partners. These partnerships are essential for improving patient safety and outcomes in surgery.

## Supplementary Information

**Additional file 1**. Terms of Reference.

## Data Availability

Not applicable.

## Abbreviation

OR: Operating room
