# Supplementary material for: Bringing the patient voice into the operating room: engaging patients in surgical safety research with the Operating Room Black Box®
Source: Res Involv Engagem. 2022 Jul 23;8:32. doi: 10.1186/s40900-022-00367-5 (PMC9308267; doi:10.1186/s40900-022-00367-5)
Supplement: Supplementary file 1 — Additional file 1. Terms of Reference. [file 40900_2022_367_MOESM1_ESM.docx]

| **Operating Room Black Box Research Program Patient Advisors**  **TERMS OF REFERENCE**  February 2018 |
| --- |

**Purpose:**

1. The Operating Room (OR) Black Box program is a research program taking place at the Ottawa Hospital.
2. Patient-advisors are important for the OR Black Box program to ensure research/quality improvement objectives and outputs reflect the lived experiences of surgical patients.
3. The patient-advisors will be invited to sit on numerous committees with clinicians and researchers to share their experiences and insights on various aspects of the project.

**Who are the patient-advisors?:**

1. Individuals who have experienced surgeries and are familiar with the healthcare system.

**Patient-advisor Responsibilities:**

1. Be available for approximately 4 hours a month to participate in meetings or teleconferences
2. Sits on *Interprofessional Clinician and Patient Working group,* which is a quality improvement (QI) working group comprised of clinicians (nurses, anesthesiologists, surgeons) and patient advisors. This group will meet quarterly to discuss how the OR Black Box can be used to meet the needs of each stakeholder.
3. Sits on *The Patient Advisory Group*, an advisory group comprised of patients, clinicians, and researchers to inform research goals, discuss findings, and advance our research program. This group will meet quarterly at The Ottawa Hospital. The research team will present current study plans, progress, and results. The material presented will be open to feedback from the advisory group.
4. Provide input on the planning and execution of the *OR Black Box Information Campaign.*
5. Notify the team’s Research Assistant if there’s any change to their availability.
6. Sign and comply with the OHRI Statement of Corporate Confidentiality.
7. Seek approval to share any ORBB information outside the research group, including through social media.
8. Other responsibilities may emerge in the future, determined in collaboration with the research team and patient-advisor.

**Research Team Responsibilities:**

1. Provide patient-advisors with resources and clear information on the project details.
2. Coordinate meeting times and prepare meeting agendas.
3. Provide patient-advisors with parking vouchers or bus tickets for transportation to meetings and/or research activities.
4. Ensure that the Terms of Reference remains relevant and amend as required.

**Communication:**

1. Communication with patient-advisors will take place through email and/or phone.
2. All meeting dates will be set up with adequate notice and will be made to accommodate patient-advisors when possible.

**Meetings:**

1. Most meetings will take place at the General hospital in the *Centre for Practice Changing Research*.
2. Meetings may occasionally take place at a different location, such as the Civic campus.
3. Either an agenda or the general purpose of the meeting will be sent to patient-advisors ahead of time.
4. Patient-advisors are welcome to bring a support person or caregiver should they feel it important to their personal wellbeing.

**Name and Contact information of the Research Team:**

**Name and Contact information of the Patient Advisor:**

Name:
Email:
Phone :

Address:
